# Supplementary material for: The Metabolomic Approach for the Screening of Endometrial Cancer: Validation from a Large Cohort of Women Scheduled for Gynecological Surgery
Source: Biomolecules. 2022 Sep 2;12(9):1229. doi: 10.3390/biom12091229 (PMC9496630; doi:10.3390/biom12091229)
Supplement: Supplementary file 1 [file biomolecules-12-01229-s001.zip › biomolecules-1845064-supplementary.pdf]

The metabolomic approach for the screening of endometrial cancer: validation from a large cohort of women scheduled for gynecological surgery

Jacopo Troisi[1,2,3*§], Antonio Mollo[1§], Martina Lombardi[2,3§], Giovanni Scala[2,4], Sean M. Richards[5,6], Steven J. K. Symes[6,7], Antonio Travaglino[8], Daniele Neola[9], Umberto de Laurentiis[1], Luigi Insabato[9], Attilio Di Spiezio Sardo[9], Antonio Raffone[10*§] and Maurizio Guida[11§]

1 Department of Medicine, Surgery and Dentistry, "Scuola Medica Salernitana", University of Salerno, Baronissi, SA, Italy
2 Theoreo srl, Via degli Ulivi 3, 84090, Montecorvino Pugliano, SA, Italy
3 Department of Chemistry and Biology, "A. Zambelli", University of Salerno, Fisciano, SA, Italy
4 Hosmotic srl, Via Raffaele Bosco 178, 80069, Vico Equense, NA, Italy
5 University of Tennessee College of Medicine, Department of Obstetrics and Gynecology, Section on Maternal-Fetal Medicine, 960 East Third Street, Suite 100, 902 McCallie Avenue, Chattanooga, TN 37403, USA
6 Department of Biology, Geology and Environmental Sciences, University of Tennessee at Chattanooga, 615 McCallie Ave., Chattanooga, TN, 37403, USA
7 Department of Chemistry and Physics, University of Tennessee at Chattanooga, 615 McCallie Ave., Chattanooga, TN, 37403, USA
8 Anatomic Pathology Unit; Department of Advanced Biomedical Sciences, University of Naples Federico II, Naples, Italy
9 Gynecology and Obstetrics Unit, Department of Public Health, University of Naples Federico II, Naples, Italy
10 Division of Gynaecology and Human Reproduction Physiopathology, Department of Medical and Surgical Sciences (DIMEC). IRCCS Azienda Ospedaliero-Univeristaria di Bologna. S. Orsola Hospital. University of Bologna, Via Massarenti 13, Bologna 40138, Italy
11 Gynecology and Obstetrics Unit, Department of Neuroscience, Reproductive Sciences and Dentistry, University of Naples Federico II, Naples, Italy

SUPPLEMENTAL MATERIAL

Table S1. Ensemble Machine Learning score for the detection of endometrial cancer in patients of the different classes divided for age subgroups (<50-year-old and ≥50-years-old)

	**<50-year-old**		**≥50-years-old**		
	EML-score Means	EML-score standard deviation	EML-score Means	EML-score standard deviation	p-value
Breast Cancer	-62,3	139,5	15,5	113,1	0,31
Cervical Cancer	81,9	208,4	93,1	179,3	0,91
Endometrial cancer	360,5	102,7	399,3	80,2	0,30
Controls	63,5	170,8	23,5	125,0	0,25
Endometriosis	129,5	202,9	30,7	161,0	0,14
Endometrial Hyperplasia	63,7	116,2	-119,0	130,6	0,06
High-grade Squamous Intraepithelial Lesion	118,5	219,6	-149,0	180,5	0,11
Low-grade Squamous Intraepithelial Lesion	31,2	167,5	41,7	160,8	0,88
Myomas and/or Polyps	65,5	163,0	43,1	162,8	0,35
Ovarian Cyst	54,5	169,7	26,5	176,2	0,43
Ovarian Cancer	75,0	200,6	112,1	233,3	0,66
Uterine Malformation	26,3	206,6	34,6	153,5	0,91
Uterine Sarcoma	116,0	100,0	178,3	4,1	0,77
Vaginal Cancer	9,0	163,9	61,5	128,5	0,55
